# The gamble between oncolytic virus therapy and IFN

**DOI:** 10.3389/fimmu.2022.971674

**Published:** 2022-08-25

**Authors:** Qingbo Li, Fengxian Tan, Yuanyuan Wang, Xiaohui Liu, Xianbin Kong, Jingyan Meng, Long Yang, Shan Cen

**Affiliations:** ^1^ College of Traditional Chinese medicine, Tianjin University of Traditional Chinese Medicine, Tianjin, China; ^2^ Research Center for Infectious Diseases, Tianjin University of Traditional Chinese Medicine, Tianjin, China; ^3^ School of Integrative Medicine, Tianjin University of Traditional Chinese Medicine, Tianjin, China; ^4^ Institute of Medicinal Biotechnology, Chinese Academy of Medical Science, Beijing, China

**Keywords:** oncolytic virus, IFN, signaling, oncology, tumor, cancer, immunology

## Abstract

Various studies are being conducted on oncolytic virotherapy which one of the mechanisms is mediating interferon (IFN) production by it exerts antitumor effects. The antiviral effect of IFN itself has a negative impact on the inhibition of oncolytic virus or tumor eradication. Therefore, it is very critical to understand the mechanism of IFN regulation by oncolytic viruses, and to define its mechanism is of great significance for improving the antitumor effect of oncolytic viruses. This review focuses on the regulatory mechanisms of IFNs by various oncolytic viruses and their combination therapies. In addition, the exerting and the producing pathways of IFNs are briefly summarized, and some current issues are put forward.

## 1 Introduction

In recent years, research on tumor treatment by oncolytic viruses has been carried out continuously, and the related mechanism studies have been explored step by step, including direct lysis of tumor cells, inhibition of tumor angiogenesis and activation of human immunity (1). Among these mechanisms, IFN, as an active component of the human immune system, always plays an essential role in the treatment of tumors (2–4). Viruses can cause changes in IFN, as can oncolytic viruses. The release of IFN activates the human immune response through various pathways, thereby reversing the immunosuppressive state of the tumor microenvironment, which plays a positive role in tumor treatment.

There is an unstable effect of oncolytic virus therapy when applied to tumor treatment, which is very closely related to the effect of IFN. On the one hand, many tumor cells have an intact IFN pathway, which will have an immune clearance effect on the oncolytic virus (5) and cannot continue to exert therapeutic effect on tumor cells. On the other hand, the IFN secretion caused by oncolytic viruses will recruit more immune cells, and the antiviral effect they play will undoubtedly be the extinction for oncolytic viruses, which eventually leads to making the therapeutic effect greatly reduced.

However, incomplete IFN pathway genes are present in some cells in cancer patients. It has been shown that nearly half of the 85 genes with methylation-dependent down-regulation after immortality are associated with IFN signal transduction (6), the deletion of these genes is more common in glioma, leukemia, and bladder cancer cells (7–9). Besides, pancreatic cancer, gastric cancer, hepatocellular carcinoma, and colon cancer all exhibit low expression of IFN receptors (10–14). In addition to tumor cells, many cancer patients have impaired IFN signaling in immune cells (15). Although these defects allow tumor cells to survive and can accelerate tumor cell proliferation, the absence of the IFN pathway allows the virus to escape from the immune system, thereby avoiding immune clearance and increasing the efficiency of viral tumor lysis (16). Therefore, clarifying the mechanism of action between oncolytic virus and IFN will provide a strategy for combining oncolytic virus with other therapies or modifying oncolytic virus. This will help us to properly deal with the relationship between IFN and oncolytic virus, which is very important to achieve enhanced anti-tumor effects.

In this review, it presents a 5-year review of the mechanisms of action of various oncolytic virus therapies associated with IFN. Beyond that, the mechanisms of IFN production and signaling are briefly introduced. This demonstrates the current level of research on oncolytic virus therapies in order to hopefully provide new ideas for future studies on the mechanisms regulating IFN. Of course, some of these issues need to be noted.

## 2 IFN generation and signals transmission

### 2.1 Generation of IFN

IFN was discovered in humans more than 50 years ago for its ability to elicit antiviral responses in cells (17). Currently, IFNs are classified into three types based on their sequences and cellular receptors, which including the IFNs of type I, type II and type III (18, 19). There are some differences in the pathways of production of different types of IFNs.

#### 2.1.1 Generation of type I IFN

The type I IFN family consists of several genetically encoded members, among which IFN-α and IFN-β are well known. In fact, they can be specifically divided into 16 species, which contain 12 IFN-α isoforms, IFN-β, IFN-ϵ, IFN-κ, and IFN-ω (20–27). The production of type I IFN is induced by pathogen-associated molecular patterns (PAMPs). These PAMPs can stimulate Toll-like receptors (TLRs) located on the cell membrane or endosomal membrane (28), or cytosolic pattern recognition receptors, including nucleotide sensors such as retinoid acid-inducible gene I(RIG-I)-like receptors (RLRs) or DNA sensors, to induce IFN production (29).

Firstly, the generation of type I IFNs is dependent on the TLR pathway. TLR recognizes double-stranded RNA and single-stranded RNA or double-stranded DNA, respectively, through TLR3, 7/8, and 9 (30), which activate and mediate IFN regulatory factor (IRF) to generate type I IFN (28, 31, 32). Second, type I IFN production can also occur through a non-TLR-dependent pathway. RIG-I and melanoma differentiation-associated protein 5 (MDA5) recognize endogenous RNA (single- and double-stranded, respectively) (33) and activate IRF3 and IRF7 to generate type I IFN through a mitochondrial antiviral signaling protein (MAVS)-dependent mechanism. In addition, endogenous cytoplasmic double-stranded DNA (dsDNA)-triggered synthesis of cyclic GMP-AMP (cGAMP) activates IFN gene stimulating protein (STING), which induces IRF3 to produce type I IFN (34).

#### 2.1.2 Generation of type II IFN

There exists only one type of type II IFN, that is IFN-γ. Diverse cells in the immune system are the primary source of its secretion, including innate-like lymphocyte populations such as innate lymphocytes (ILC) and natural killer (NK) cells, and also adaptive immune cells consisting of T helper 1 (Th1) cells and CD8 cytotoxic T lymphocytes (CTL) (35).

First of all, in innate lymphocytes, microbial infection or tissue injury activates pattern recognition receptors (PRR) as well as broadly reactive antigen receptors, which induce IFN-γ production. In addition, cytokines which consisting of interleukin (IL)-12 and IL-18 can also lead to IFN-γ production. Second, in adaptive immune cells, T-cell receptor (TCR)-mediated recognition of microbial peptides causes sustained high levels of IFN-γ production in Th1 cells and CTL. However, the mechanism of IFN-γ production differs between the two cell types, with Th1 cells producing IFN-γ associated with major histocompatibility complex (MHC) Class II molecules, whereas CTL production of IFN-γ is associated with MHC Class I molecules (35).

#### 2.1.3 Generation of type III IFN

Type III IFN was discovered later (known as IFN-λ) and it was reported in 2003 for the first time (36, 37). It includes four kinds of IFN-λ isoforms, namely IFN-λ1 or IL-28a, IFN-λ2 or IL-28b, IFN-λ3 or IL-29, and IFN-λ4 (38–40). Similarly, viruses can mediate the expression of type III IFNs in diverse cell types (41–43).

Type III IFN is expressed in various primary human cells of the hematopoietic spectrum (44–48), in parallel with the production of large amounts of type I IFN. Meanwhile, type III IFN is mainly produced by epithelial cells in non-hematopoietic cells (49–51). However, the exact mechanism by which it produces is not clearly explained. It has been claimed that the HSV molecular pattern is distinguished by TLR3 and TLR9 in the endosome as well as melanoma differentiation-associated gene 5 (MDA5) in the cytoplasm, which leads to the activation of nuclear factor κB (NF-κB), IRF3 and IRF7 transcription factors and their subsequent translocation to the nucleus, where they then stimulate IFN-λ gene transcription (52). In this process, the transcriptional mediator Med23 and anchoring protein repeat domain protein 1 (ANKRD1) target IRF7 and IRF3, individually, which promote the gene of type III IFN expression (53, 54).

### 2.2 Signal transduction of IFN

**Figure 1:**
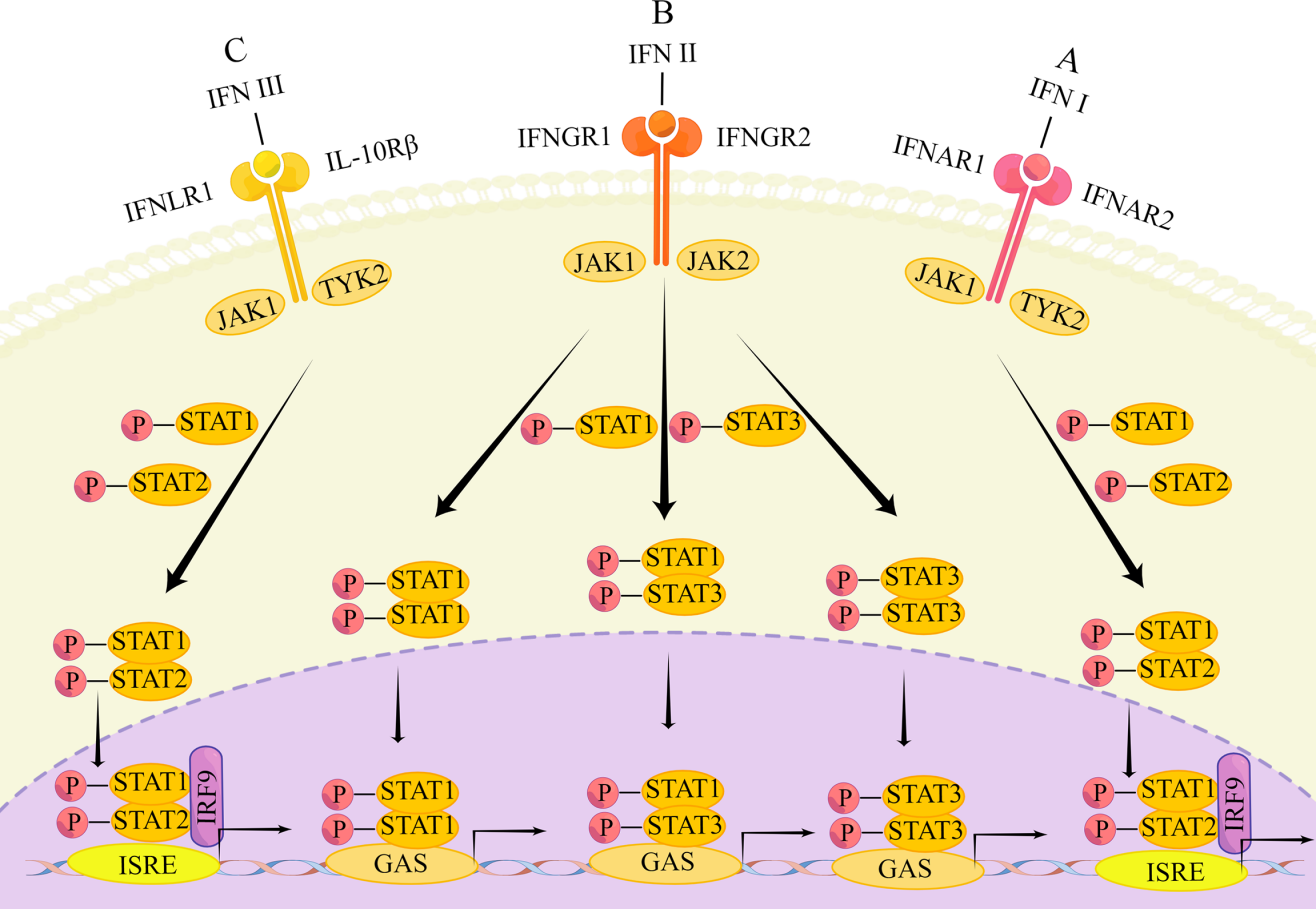
The main transduction pathway of IFN signaling. **(A)** IFN first binds to the heterodimers of IFNAR1 and IFNAR2, causing phosphorylation of JAK1 and TYK2, followed by phosphorylation of STAT1 and STAT2. Phosphorylated STATs form heterodimers that enter the nucleus and bind to IRF9 to form ISGF3, which subsequently binds to the transcriptional enhancer ISRE, triggering transcriptional induction of ISG. **(B)** Type II IFNs first bind to IFNGR1 and IFNGR2, causing phosphorylation of STAT1 and STAT2. The phosphorylated STAT forms a heterodimer that enters the nucleus and binds to IRF9 to form ISGF3. ISGF3 subsequently binds to the transcriptional enhancer ISRE, triggering the transcriptional induction of ISG. In addition, phosphorylated STAT1 homodimers, STAT3 homodimers and STAT1-STAT3 heterodimers enter the nucleus. These dimers bind to GAS elements to induce transcription factor production and initiate a second wave of gene expression; **(C)** Type III IFN binds to IFNLR1 and IL10R2, followed by the same response as the type I IFN signaling cascade.

When IFN acts, it is transduced through different pathways. The three IFNs mainly signal through the Janus kinase/signal transducer and activator of transcription (JAK/STAT) pathway. There are both similarities and differences. The main differences are that the signaling of the three IFNs is carried out through the binding of different heterodimeric receptor complexes (20) **(** **Figure 1** **)**.

Type I IFN is bound to a heterodimer of type I interferon α/β receptor 1 (IFNAR1) and IFN-α/β receptor 2 (IFNAR2) (18, 55). Signaling through the JAK/STAT pathway is the most common, which starts with phosphorylation of JAK1 and tyrosine kinase (TYK)2 and leads to phosphorylation of STAT1 as well as STAT2. Phosphorylated STATs are able to form heterodimers. The heterodimers will enter the nucleus and link with IRF9 to create the transcriptional activator IFN-stimulated gene factor (ISGF) 3. Next, ISGF3 integrates with the transcriptional enhancer called IFN-stimulated response element (ISRE), leading to transcriptional induction of ISGs (56–59). Besides this typical pathway, type I IFN can induce the expression of other genes, through STAT1 or STAT3 homodimers as well. For instance, homodimers formed by STAT1 combined with gamma activated sequence (GAS) elements that belong to different genes’ promoters (60). STAT1 and STAT3 are the most frequent, but it has also been shown that in some cell types, STAT-3, -4, -5, and -6 can also be activated by interferon receptor (IFNAR), causing the next series of signaling cascades (61).

Type II IFN is bound to type II interferon gamma receptor 1 (IFNGR1) and IFN-γ receptor 2 (IFNGR2) (18, 55). The JAK/STAT pathway can be activated as well, although not in the same way as the changes in the JAK/STAT pathway caused by type I IFN. In this pathway, type II IFN can signal through ISGF3 as type I IFN does (62), causing the following series of responses. The difference is that the phosphorylation of JAK2 upon binding of type II IFN to its receptor is accompanied by allowing phosphorylation of JAK1 and IFNGR1 (63), which will recruit and phosphorylate STAT1s. Then, the homodimer formed by phosphorylated STAT1 enters the nucleus, unites with the GAS element to induce transcription, inducing the generation of many transcription factors that initiate the second wave of gene expression (64). Furthermore, IFN-γ signaling can activate not only STAT1 homodimers, but also generate STAT3 homodimers and STAT1-STAT3 heterodimers. These still translocated to the nucleus to combined with the GAS element that is in the IRG gene promoter (65, 66). Apart from the above pathway, type II IFN can trigger the expression of MHCII as well, that is by inducing a different piece of genes through the function of the class II, major histocompatibility complex, transactivator (CIITA) (67).

Type III IFN is bound to interferon λ receptor 1 (IFNLR1) and IL-10 receptor 2 (IL10R2) (also known as IL-10 receptor β (IL10Rβ)) (18, 55). The induced signaling pathway is also essentially the same as that of type I IFN (36). Type III IFNs similarly form ISGF3 through phosphorylated STAT1 and STAT2, followed by binding to IRF9, which in turn triggers the expression of ISGs (42). Alternatively, type III IFN can induce the activation of STAT-3, -4, and -5 in certain cell types (68). However, the durability of ISG induction by type III IFN is demonstrated by the fact that ISGs peak later after type III IFN stimulation and persist over time. In contrast, type I IFN only induces ISG expression at an early stage and persists for a relatively short period of time (69).

Aside from the classical pathway of JAK/STAT, the three IFNs can also function in other signaling pathways, including MAPK and PI3-kinase pathways (61, 70). In addition, type I IFN can also activate and signal through the NF-κB pathway, and type II IFN can function through this pathway as well (70). Also, the bioinformatic analysis revealed the presence of NF-κB binding sites in the promoter of the type III IFN gene (68), suggesting the possibility that it also acts in the NF-κB pathway.

## 3 IFN and tumor treatment

IFN is able to modulate multiple pathways to achieve tumor inhibition or killing. This has been demonstrated in several experimental studies.

Under *in vitro* conditions, IFN can inhibit tumor growth through various pathways. First, IFN can affect the cell cycle of tumor cells. For example, IFNα can arrest the cell cycle of prostate cancer cells, which is achieved by upregulating endogenous inhibitors of cell cycle protein-dependent protein-dependent kinases, such as p21 (71); it has been discovered that type I IFNs can prolong the cell cycle of human breast cancer cells under *in vitro* conditions, which can suppress the growth of tumor cells (72). Second, IFN can also induce apoptosis of tumor cells. It has been shown that type I IFNs and Toll-like receptor 3 (TLR3) agonists when combined, are able to upregulate DR ligands, tumor necrosis factor-related apoptosis-inducing ligand (TRAIL), thus leading the breast tumor cell lines to be apoptotic (64). Moreover, type I IFN-induced apoptosis was connected with other ISGs, consisting of Fas, Fas ligand (FASLG), protein kinase R (PKR), and 2′-5′-oligoadenosine synthase (OAS) (64).

In the vivo environment, IFNs have been suggested to have a crucial role in tumorigenesis process. For example, in studies of 3-methylcholanthrene (MCA)-induced sarcoma models, it was found that deletion of immune cell type I and/or type II IFN signaling pathways sped up tumorigenesis and development (73, 74). Furthermore, STAT1 is thought to exert antitumor effects in transgenic mouse models of breast cancer through activation of immune and antiproliferative mechanisms (75), which is significant in type three types of IFNs signaling.

## 4 Molecular mechanisms by which different oncolytic virus therapies affect the IFN pathway

### 4.1 Vesicular stomatitis lysis virus

**Figure 2:**
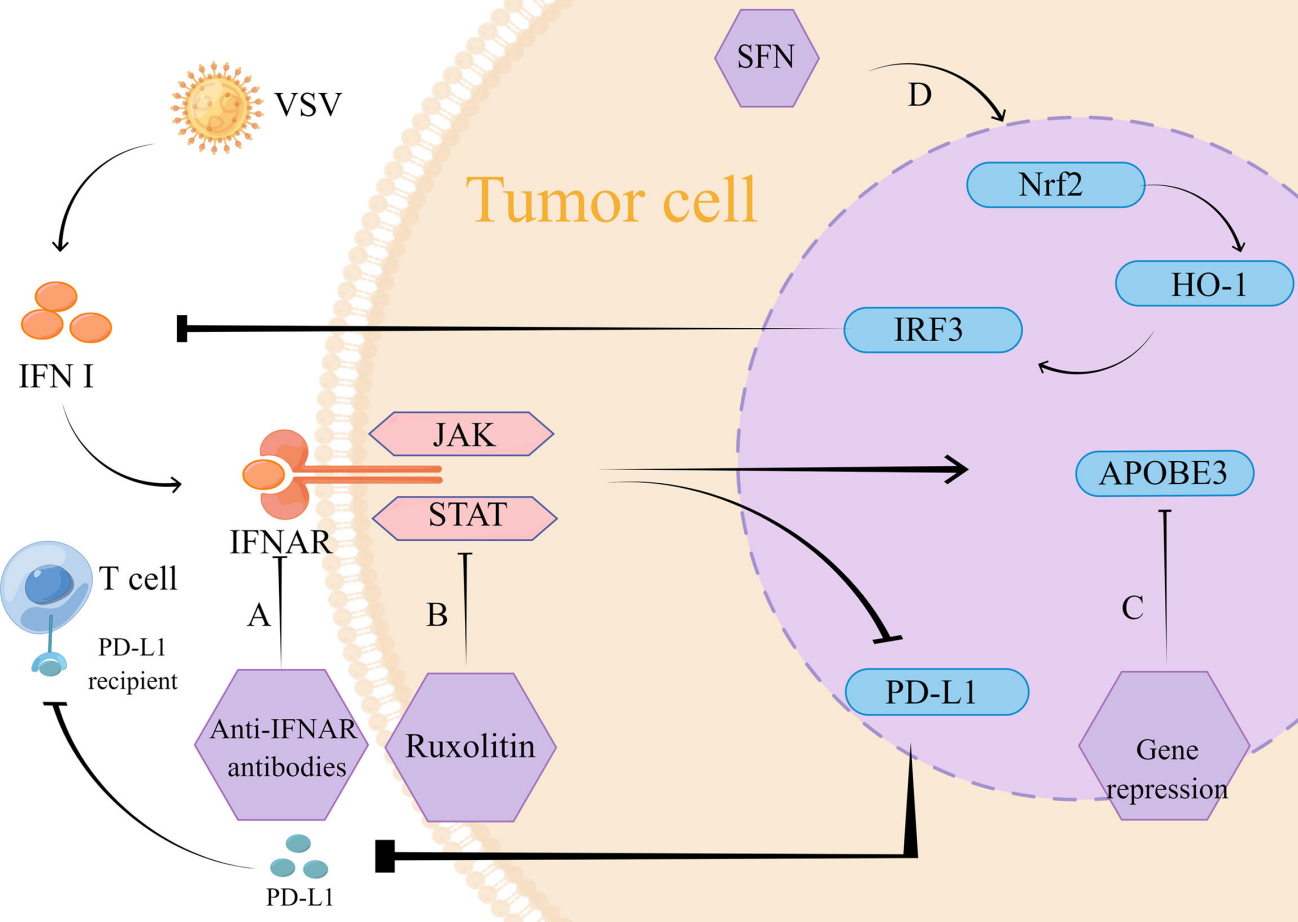
Mechanism of action of VSV therapy. **(A)** The intervention of monoclonal IFNAR antibody blocks the VSV-mediated IFN inflammatory pathway and reduces type I IFN-induced PD-L1 expression. Its low expression reduced PD-L1 binding to T cells, which thus exerted normal antitumor effects. **(B)** VSV strongly induced the JAK/STAT pathway, and inhibition of JAK/STAT using lusolidin prevented IFN-mediated antiviral response to VSV immune clearance, which promoted VSV replication and dissemination. Similarly, type I IFN-induced PD-L1 expression was reduced, thus preventing PD-L1 from binding to T cells and providing conditions for T cells to exert normal anti-tumor effects. **(C)** APOBEC3 gene expression is mediated by type I IFN, targeted inhibition of APOBEC3 gene can reduce IFN-mediated tumor resistance. **(D)** SFN through Nrf2/HO-1 pathway activates autophagy to inhibit IRF3 activity, which suppresses the type I IFN response. The inhibition of IFN response reduces the restriction of replication of VSVΔ51, so VSV exerts its normal oncolytic effect.

VSV is a prototypical member of the genus Blister virus, belonging to the family Rhabdoviridae (76). Recently, it has been extensively studied as an oncolytic agent (77). Among the IFN-related mechanisms, VSV mainly regulates IFN-induced antiviral factors, the expression of classical JAK/STAT, nuclear factor red lineage 2-related factor 2 (Nrf2), and IFN-mediated programmed cell death-ligand 1 (PD-L1) **(** **Figure 2** **)**.

The type I IFN response is considered as an essential pathway for the development of drug resistance to VSV during tumor treatment, and tumor cells with intact or partially intact IFN signaling are resistant to viral replication (78–80). Human A375 as well as mouse B16-OVA melanoma cell lines were reported to be shielded by type I IFN and resistant to tumor lysis by wild-type VSV and VSV-GP (81)., thus preventing VSV from exerting its normal antitumor effects. VSV significantly upregulates the JAK/STAT pathway, which is an important component of the functioning of IFN. Inhibition of this link can effectively improve resistance to VSV therapy. In a preclinical trial in small cell lung cancer, the use of the JAK/STAT inhibitor lusolidin effectively increased viral replication, and the killing effect of VSV-IFN-β on tumor cells was enhanced *in vitro* conditions. Besides, it turned out that PD-L1 expression was restricted, which was also beneficial for tumor treatment. However, this combination treatment strategy did not significantly improve the survival rate of mice (82), so the safety of this combination therapy is uncertain. Additionally, this pathway was also studied in an animal experiment in melanoma, where tumor sensitivity to VSV-Δ51 was significantly increased under conditions of JAK/STAT pathway inhibition (83).

PD-L1 performs a functional role in regulating the cancer immune clearance cycle by binding to T cell-activated negative regulators, such as programmed cell death-1 (PD-1) and B7.1 (CD80) (84). In order to inhibit the killing effect on tumor cells, the combination of PD-L1 and its receptor inhibits T cells from migrating, meanwhile, the combination keeps down the T cells’ proliferation as well as the release of mediators that have cytotoxic, therefore inhibition of PD-L1 expression is one of the strategies for tumor therapy. Several previous studies have shown that PD-L1 expression can be induced independently of the IFN inflammatory pathway, but is often dependent on the IRF1 pathway, a transcription factor associated with PD-L1 regulation (85–88). In contrast, according to a recent research in melanoma, VSV optimizes PD-L1 upregulation in tumors which is dependent on type I IFN expression, and in-depth studies revealed that VSV-induced type I IFN proceeds in an IFNAR-dependent manner (89). This provides an opportunity to improve the therapeutic use of VSV for tumors. In order to block the viral-mediated IFN inflammatory pathway, the monoclonal IFNAR antibody can be taken into consideration. The paper shows monoclonal IFNAR antibody can reduce PD-L1 expression which is induced by type I IFN, this will promotes immune responses with tumor-specific T cells (89). This research result provides a new target for the treatment of solid tumors.

Among the IFN-induced antiviral factors, the APOBEC cytosine deaminase family is associated with viral resistance (90), which has been demonstrated in retroviruses, herpesviruses, and hepatitis viruses, among others (91–94). In addition to being a viral limiting factor, over expression of APOBEC3 family proteins occurs in several types of cancers, so that APOBEC3 upregulation and the genomic mutations it causes to mediate therapeutic resistance are important for the prognostic profile of cancer (92, 95). In contrast, VSV, a retrovirus, is able to mediate APOBEC3 expression in tumor cells. This expression is dependent on type I IFN upregulation. This research suggests that APOBEC3 is a key gene for type I IFN stimulation and plays an influential part in the build-up of resistance to oncolytic virus therapy (96).

Nrf2 is a transcriptional regulator that maintains redox homeostasis by controlling basal and induced expression of a series of antioxidant enzymes (90). Furthermore, Nrf2 actively regulates autophagy as an essential component of the regulatory network that responses to different types of stress, including protein aggregation, nutritional deficiency, and viral infection (97). Therefore, it may also influence VSV replication and infection. A study on lung cancer and osteosarcoma showed that for drug-resistant lung cancer cells (A549) and osteosarcoma cells (U-2OS), sulforaphane (SFN) inhibited IRF3 activity by activating autophagy through the Nrf2/HO-1 pathway. This inhibited the type I IFN response and promoted VSVΔ51 replication, leading to better tumor lysis. what’s more, it has shown a good safety profile in animal experiments (98).

### 4.2 Herpes simplex virus-1

**Figure 3:**
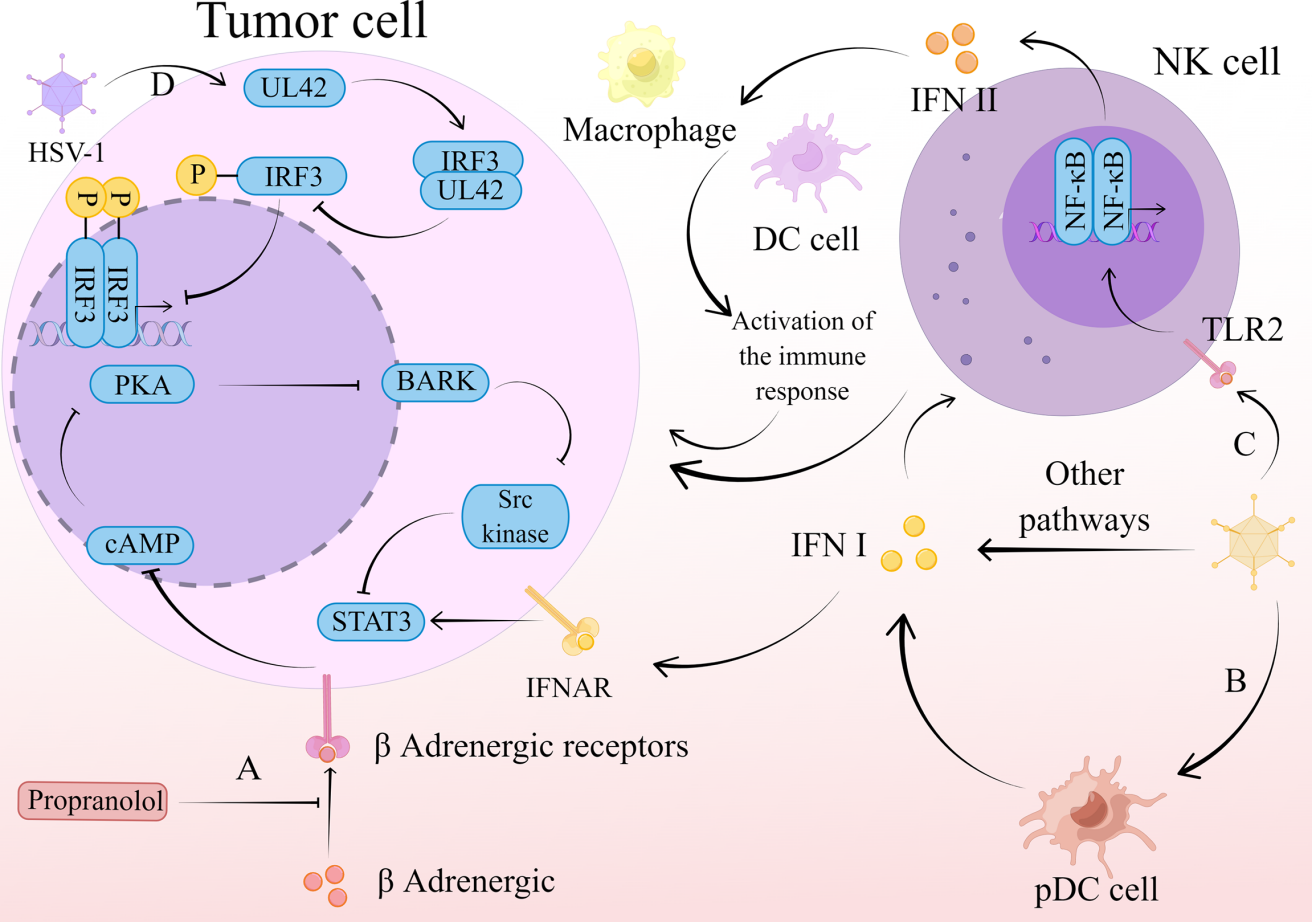
The mechanism of action of HSV-1 therapy. **(A)** Propranolol blocks β-adrenergic binding to β-adrenergic receptors, leading to a restriction of Gαs-mediated cAMP synthesis. This restriction limits the transient flux of intracellular cAMP to activate PKA production and inhibits its phosphorylation to produce BARK. activation of Src kinase is dependent on BARK, and its inhibition in turn inhibits activation of the transcription factor STAT3. This series of reactions inhibit the type I IFN-mediated antiviral response and promotes the normal replication and propagation of HSV-1, thus exerting an oncolytic effect. **(B)** HSV-1 stimulates the production of type I IFN by pDC, which activates NK cells to exert a direct killing effect on tumor cells. **(C)** HSV-1 stimulates the release of type II IFN by NK cells through the TLR2/NF-κB signaling pathway. Type II IFNs released by NK cells recruit macrophages, DC cells, and other immune cells, which act as immune agents to remove tumor cells. **(D)** The viral polymerase synthesis factor UL42 is able to interact with the host transcription factor IRF3. This interaction inhibits IRF3 phosphorylation and downstream IFN-β gene transcription, thereby suppressing IFN-β expression and thus the antiviral effect. The virus is consequently able to replicate and spread normally, exerting an oncolytic effect.

Herpes simplex virus 1 (HSV-1) belongs to the subfamily Alphaherpesvirinae (99). Regulation of IFN by HSV and its combination therapies is achieved through multiple pathways, including STAT3-PKR-dependent antiviral responses, IRF3, and TNF-related apoptosis-inducing ligand (TRAIL). Remarkably, there is a possibility as a potential cancer vaccine of it. **(** **Figure 3** **)**

Similarly, HSV still faces resistance due to innate immunity when applied. The type I IFN antiviral signaling pathway bears the brunt of viral defense in infected cells (100). Previous studies have identified protein kinase R (PKR) as a host antiviral kinase that inhibits cell proliferation and blocks the production of viral proteins, thus preventing viral replication (101). Therefore, inhibition of its expression and activation can attenuate the type I IFN-mediated antiviral response. In contrast, STAT3, as part of the type I IFN signaling pathway, can also inhibit the expression of PKR to limit the type I IFN cascade response (102–105). Similarly, protein kinase A (PKA) can also achieve this effect. Recently, it has been shown that β-blocker pretreatment inhibits the binding of catecholamines to β-adrenergic receptors, leading to a limitation of Gαs-mediated cyclic 3’-5’ adenosine monophosphate (cAMP) synthesis. This limits the transient flux of intracellular cAMP to activate PKA production, whose phosphorylation to produce a variety of target proteins is necessarily affected, including β-adrenergic receptor kinase (BARK). Src kinase can be activated by BARK, thereby suppressing the activation of the important transcription factor STAT3 (106). By inhibiting this series of interferon-related responses, the antitumor efficacy of oncolytic virus T1012G is improved (107).

The IFN gene stimulating factor (STING), which is upstream of IFN production, is similar to STAT. Its mediated IFN gene stimulating factor (STING)- TANK-binding kinase 1 (TBK1)-IRF3-IFN pathway is a central cellular host defense against viral infection (108–110). However, in a preclinical study of pancreatic ductal adenocarcinoma (PDAC), it was shown that cell lines defective in the STING pathway had relatively low susceptibility to being C-REV (a type of HSV) (111). In contrast, those cell lines that capable of responsive STING pathway had relatively higher susceptibility to C-REV. This suggests that there is a correlation between STING pathway activation and resistance to C-REV, and this pathway does have an effect on oncolytic virus replication, However, data analysis revealed that it is not the main pathway affecting C-REV in human pancreatic ductal adenocarcinoma cell lines (111).

Although C-REV has no critical effect on STING, one study found that modification of HSV can act on IRF3 in the STING pathway to exert antitumor effects. Engineered HSV-1ΔN146 containing amino acids 147 to 263 of γ134.5 could efficiently replicate and lyse in malignant cells refractory to the γ134.5 null mutant. ΔN146 activated IRF3 and IFN expression, triggering immunity against the virus and the tumor. Unexpectedly, ΔN146 exposed to exogenous IFN-α was also able to replicate normally, and in a 4T1 tumor model, ΔN146 also exhibited significant inhibition of tumor growth and metastasis. Thus, ΔN146 is able to stimulate the expression of inflammatory cytokines that do not have a serious impact on the replication of the virus in tumor cells. This can ensure to a greater extent that the oncolytic virus works (112). Furthermore, a similar effect can be achieved by causing mutations in the HSV-1 gene through the random insertion of a corruptive 1.2-kbp transposon into the viral genome. This mutation is capable of generating the viral polymerase synthesis factor UL42, which interacts with the host transcription factor IRF3 (113). The above studies suggest that genetic modification of HSV may be a new strategy to avoid immune clearance of HSV and provide a new way to enhance the effect of oncolytic virus therapy, but this may only be applicable to personalized treatment.

One of the pathways mediated by HSV-1 to generate IFN-α/β is through the stimulation of plasmacytoid dendritic cells (pDCs) (114), whose production of type I IFNs can activate NK cells (115). A current study has found that the type I IFN produced by pDCs and activated NK cells are an important link in the anti-myeloma effect of HSV-1, type I IFN has a direct cytotoxic effect on tumor cells and induces IFN to release from NK cells, thereby enhancing the killing effect of NK cells (116). In addition, type I IFNs have direct oncolytic activity against plasmacytoid tumors, where type I IFNs upregulate TRAIL expression, mitochondrial cytochrome c release, while limiting the expression of B-cell leukemia/lymphoma 2 (Bcl-2) and Bcl-XL (117). Regulation of these genes ultimately leads to apoptosis.

HSV not only acts through type I IFNs with NK cells, but also mediates type II IFNs stimulation of NK cells. Recent studies have shown that UV-oHSV2 can stimulate NK cells to secrete IFN-γ, which is achieved through the Toll-like receptor 2 (TLR2)/NF-κB signaling pathway, which activates multiple immune cells to exert anti-tumor responses (118). Furthermore, UV-oHSV2 stimulation promoted the expression of two checkpoint molecules, one located on NK cells, NKG2A, and the other on tumor cells, HLA-E. This finding predicts that anti-NKG2A may further enhance the antitumor effects occurring from UV-oHSV2 stimulation, and that anti-HLA-E treatment also has this possibility (118).

Speaking of IFN-γ, it plays an active role in the induction of apoptosis as well as tumor-infiltrating T cell recruitment. It has been shown that oHSV-1 stimulates tumor cells to secrete IFN-γ, which increases the immune activity of T cells, which is also able to enhance the activity of CD70-specific CAR T cells. This combination of specific T-cell therapy and oHSV-1 enhanced the pro-inflammatory environment and reduced anti-inflammatory factors *in vitro*, which achieved the goal of promoting tumor extinction. While this immune activation environment, this combined strategy increased the ratio of T cells and natural killer cells in the tumor microenvironment (TME) and decreased the expression of regulatory T cells as well as transforming growth factor-β1 in glioblastoma (GBM) in an *in situ* xenograft animal model. It undoubtedly brings a new therapeutic strategy for the treatment of GBM (119).

In addition, one study found that HF10 was able to prevent secondary tumors while activating anti-tumor effects. In an animal experiment with mice with squamous cell carcinoma, mice that survived HF10 treatment all showed rejection of tumors upon reactivation. Studies of systemic immunity in mice revealed that a large number of granulocytes and CD8 T cells were accumulated in the spleen at the time of HF10 application, and when co-cultured with SCC-VII cells, splenocytes released type I IFN (IFN-α and IFN-β), IFN-γ, IL-2, IL-12, and TNF-α. This suggests that mice developed anti-tumor immunity and implies that HSV has the potential for an *in situ* cancer vaccine (120).

### 4.3 Reovirus

MRV is a virus with double stranded RNA (dsRNA), which belongs to Reoviridae (121). Its mechanisms associated with IFN when treating tumors are mainly related to the PD-1/PD-L1 axis, STAT, and IFNAR signaling.

Similar to VSV, IFN is a key cytokine in reovirus-mediated activation of immune cell populations (122). A research found that type I and type II IFNs are able to promote PD-L1 expression in patient-derived glioma cells in a synergistic manner, while type I IFNs can induce strong expression of PD-L1 with IFN-γ, which undoubtedly has a detrimental effect on tumor treatment (123). PD-L1 binding to PD-1 prevents attack by the host’s own immune system, thereby reducing T-cell activation and proliferation of cytotoxic T lymphocytes. tumor cells evade immune surveillance as a result of T-cell depletion (124, 125). In a vitro experiment, IFN-γ was the cytokine secreted after reovirus treatment of HGG cells (123). The above analysis revealed that reovirus treatment could improve the clinical outcome of brain tumor patients by activating leukocytes, enhancing T-cell infiltration into tumors and upregulating PD-L1, which prepared for later anti-PD-1 therapy. Further studies found that the addition of PD-1 blockers to reovirus enhanced systemic therapy in preclinical glioma models. These results analyze the mechanisms by which reovirus affect IFN-related pathways and provide theoretical support for the development of PD-L1 blockade combined with systemic immunoviral treatment strategies (123).

It has been noted that in a mouse model, IL-15 can be induced by type I IFN in dendritic cells (DC production) and this cytokine can activate NK cells to act (126, 127). NK cells are a type of innate lymphocyte (ILC) that, on the one hand, are able to recognize and kill infected cells as well as tumor cells, on the other hand, have an ability to induce adaptive immunity to function (128, 129).NK component of the cytotoxic machinery is triggered by type I IFN (126, 130, 131). Recent studies have shown that Reovirus are able to activate NK cells in a type I IFN -dependent manner, inducing STAT1 and STAT4 signaling in CD56^dim^ as well as a subset of CD56^bright^ NK cells. However, It is puzzling that MRV is dependent on type I IFN to inhibit IL-15-induced NK cell proliferation, which may be involved with reduced AKT signaling. In *in vivo* experiments, CD56^bright^ NK cells disappeared from the peripheral circulation for a brief period during the peak of the type I IFN response, which may suggest that they underwent redistribution and migrated to secondary lymphoid tissues. In combination with OV-mediated direct tumor cell killing, CD56 activation and CD56^bright^ NK cells induce a spectrum of activity *via* antiviral pathways, including NK cell-mediated tumor cell killing and regulation of adaptive NK cells to lymph nodes by transport of IFN-γ-expressing CD56 (132). However, reovirus does not always depend on the type I IFN pathway for its antitumor effects. By comparing IFN-β promoter stimulator-1promoter stimulator-1 knockout (KO) mice with TLR-3 KO mice under reovirus treatment conditions, it was found that Reovirus inhibits the immunosuppressive activity of bone marrow mesenchymal stem cells in a TLR3 manner, but not in an IFN-β promoter stimulator-1 signaling-dependent manner (133).

Alternatively, the type of IFN produced by reovirus-mediated production can impact the tumor’s therapeutic outcome. Studies have shown that MRV infection has a superior stimulatory effect on type III IFN production, but does not show satisfactory performance for type I IFN production. Although activation of STAT1 and STAT2 can be achieved by both type I and type III IFNs, triple-negative breast cancer cell proliferation is only sensitive to type I IFN (134). For this issue, researchers treated triple-negative breast cancer cells with a topoisomerase inhibitor that activated the DNA damage response pathway. This combination promoted the replication of the eutherian virus and enhanced cytotoxicity, achieving effective infection and killing of triple-negative breast cancer cells (134).

Again, the problem of ineffectiveness against IFN pathway-deficient tumor cells has been faced with the application of the eutherian virus. Researchers identified IFN regulatory factor 3, as a crucial transcription factor for IFN-β expression, in transformed human myeloid cells infected with tumor-selective MRV, IFN-α/β receptor (IFNAR) signaling both gradually promoted IFN I secretion from infected cells by enhancing the activation of IFN regulatory factor 3, and also promoted viral replication. However, tumors can interfere with the IFNAR pathway to maintain their own survival, and tumors that do not respond to IFNAR signaling may require other therapeutic strategies to promote adequate type I IFN secretion into the tumor microenvironment. Therefore, the parameters of eutherian virus-induced type I IFN levels need to be further explored (135).

Some viruses from the same ancestor have small genetic differences that cannot be ignored, and their effects on cell signaling and regulation of cytokine secretion may differ dramatically (136–142), which can affect antitumor effects. For example, T3D lab strains have a large variability in the regulation of RIG-I and/or IFN-dependent genes, with the least tumorigenic T3DTD strains inducing significantly higher levels compared to the most tumorigenic T3DPL strains (the difference may be a result of polymorphisms in the dsRNA-binding protein and the PKR antagonist σ3), which is crucial for the selection of the appropriate tumorigenic virus strain (143). This suggests the need to consider minor differences between viruses and to clarify the target of action when selecting combination therapies.

### 4.4 Newcastle disease virus

NDV belongs to the genus Aviravirus in the family Paramyxoviridae (144). The mechanism has not been extensively studied in terms of IFN-associated tumor lysis, which is associated with both type I and type II IFNs.

Similar to numerous viruses, NDV affects type I IFN (145). It is well known that type I IFN-mediated PD-L1 expression is an unfavorable factor in tumor treatment. Unexpectedly, the inflammatory response and PD-L1 upregulation induced by NDV treatment of tumors can precisely enhance the sensitivity of these tumors to PD-1/PD-L1 blockade. The strategy of intratumoral NDV injection combined with systemic PD-1 or PD-L1 blockade significantly enhances the antitumor immune effect, which provides a theoretical basis for future clinical trials (146). Further analysis revealed that this is mainly evidenced by its upregulation of PD-L1 expression in tumor cells as well as in tumor-infiltrating immune cells, which plays an important role in the development of late adaptive mechanisms of immune resistance to increased immune cell infiltration into tumors (146).

NDV infection also affects changes in type II IFN, as demonstrated in glioblastoma, colorectal and cervical cancers (147–149), and the mechanism of action needs to be further elucidated. In an animal experiment on lung cancer, it was identified that, compared to IL-4, the increase in IFN-γ concentration far exceeded its increase, IFN-γ is one of the cytokines secreted by Th1, under NDV intervention conditions (150). This indicates a shift in cellular distribution from Th2-dominant to Th1-dominant, suggesting that NDV plays a role in regulating humoral immunity and inhibiting tumor growth (148).

Naturally, genetic modification of NDV is one of the strategies to overcome the body’s antiviral reflection (151). It was found that genetic modification of NDV to express influenza virus NS1 protein can improve the susceptibility of GBM to type I NDV-activated cells, resulting in better tumor lysis (152). This viral protein can suppress the host immune response (153, 154), the virus can thus exert its antitumor effects more effectively.

### 4.5 Vaccinia virus

The virus of VV belongs to the genus Orthopoxvirus (OPXV) (155). Its mediated IFN exerts antitumor effects mainly related to IRF-3, JAK-STAT signaling pathway, and Th1 cells.

In the past decades, some progress has been made in the study of VV for antitumor therapy (156–159). The finding of a recent study that a recombinant VV can induce high levels of IFN while blocking the IFN-mediated antiviral response is undoubtedly an important finding. It was shown that OncoVV-WCL could achieve induction of high levels of type I IFN expression by promoting IRF-3 transcriptional activity, which could enhance the antitumor effects of oncolytic virus. Specifically, blocking the IFN-induced antiviral response is achieved through two pathways. On the one hand, OncoVV-WCL can inhibit the activity of IFN stimulatory response element (ISRE), and on the other hand, inhibition of JAK-STAT signaling pathway by OncoVV-WCL limits ISG expression (160). By these means, the virus can avoid elimination by the antiviral pathway and thus exert a normal lytic effect (160).

Another study on multiple tumors showed that vvDD-IL-23 can promote the expression and release of Th1 chemokines and some anti-tumor factors, which contained IFN-γ, as well as tumor necrosis factor-α (TNF-α), IL-2, perforin, and granzyme B (GzmB). These cytokines keep the ratio of infiltrating activated T cells CD8 and Treg to high levels, which play a therapeutic role in reversing the immunosuppressive state to achieve antitumor (161). In a clinical study, VV also acted through a similar mechanism. It was found that a classical IFN response, including the release of inflammatory cytokines/chemokines, was induced in patients who were lysing virus responders. These factors activate T cells, which can then infiltrate into the tumor to exert antitumor effects (162).

### 4.6 Other viruses

#### 4.6.1 Measles virus

MV is a type of negative-stranded RNA virus that belongs to the family Paramyxoviridae, genus Morbillivirus (163). It mainly affects type I IFN to exert antitumor effects. A variety of malignant pleural mesothelioma (MPM) cell lines have a defect in antiviral type I IFN response (164), and this defect in type I IFN response is located upstream of the IFNAR. It was thought that type I IFN-deficient tumor cells sensitive to the antitumor effects of MV still participate in part of the type I IFN response though. This is achieved by relocalizing IRF3 and NF-κB in the nucleus, but the resulting ISG expression is minimal, which is very favorable for the oncolytic virus to function. Indeed, type I IFN-deficient tumor cells can induce a response that induces immunogenic death of tumor cells and additionally induces an endoplasmic reticulum stress response, enhancing the antitumor effect. At a deeper genetic level, pure deletion (HD) of all genes of type I IFN in human MPM cells leads to their sensitivity to MV virus, and HD of the type I IFN-encoding gene in MPM occurs frequently together with HD of the CDKN2A gene, it suggests a new therapeutic target (165–167). Another study using a sequential transformation model also identified reduced type 1 IFN pathway function as a significant factor in MV-mediated selectivity of transformed cytolytic tumors (168).

#### 4.6.2 Coxsackie virus

Fewer studies have been conducted on the mechanisms by which coxsackieviruses exert tumor lysis. Recent clinical trials in non-muscle invasive bladder cancer have identified a kind of coxsackievirus, CAVATAK, that upregulates PD-L1 and lymphocyte activation gene-3 (LAG3) among the IFN-inducible genes. In parallel, this virus promotes the release of Th1-related chemokines as well as induces RIG-I. Through these pathways, it induces an inflammatory response, reverses the “cold” tumor state, has an anti-tumor effect, and shows a good biosafety profile (169).

#### 4.6.3 Poliovirus

The neurally attenuated recombinant poliovirus PVSRIPO has also been used in oncology treatment, it has shown good efficacy in clinical trials in glioblastoma (170). Recently, researchers have explored its mechanism of action, and unlike other oncolytic viruses, PVSRIPO is insensitive to both upstream and downstream endogenous intrinsic responses to IFN triggered by MDA5 under *in vitro* conditions. Although the involvement of PRR inhibited the kinetics of PVSRIPO, PVSRIPO could still be translated in diseased cells and propagate in the cell. This occurrence may be related to the translation strategy of PVSRIPO, which prevents the body’s antiviral immune response while destroying tumor cells, and immune escape occurs (171). This property could sustain activation of the IFN response. This finding suggests that poliovirus has a significant advantage in exerting its oncolytic effect.

## 5 Discussions and challenges

From the above review, we can understand that a variety of viruses show the ability to activate the immune of body response, which is closely related to the IFN pathway. They reverse the immunosuppressive state in the tumor microenvironment, resulting in the production of various other cytokines and various immune cells and changing cold tumors to hot tumors. Finally, they achieve the suppressive and clearing effect on tumor cells.

For different oncolytic viruses, the pathways affecting IFN are not identical, and almost all IFN-related pathways are included, summarizing that they mainly interact with IFN through the following mechanisms: (1) PD-L1/PD-1; (2) JAK/STAT signaling pathway; (3) APOBEC cytosine deaminase family; (4) Nrf2; (5) TLR2/NF-κB signaling pathway; (6) IRF3. The elucidation of these mechanisms provides a theoretical basis for future combination therapies with various oncolytic viruses, thus providing guidance for targeted enhancement of oncolytic viral therapeutic efficacy.

At the same time, we found that oncolytic viruses are similar to other viruses. When the immunity of body is activated, it will activate the anti-virus response related to IFN, which will make the virus unable to exist in the immune microenvironment for a relatively long time. Although this can improve the biological safety of oncolytic virus therapy, it is difficult to achieve an effective anti-tumor effect for a short time. Through various ways, it can inhibit the immune clearance of oncolytic viruses, better therapeutic effect can be achieved. Here, we focus on IFNs. By summarizing, we found that researchers mainly take the following ways to achieve better anti-tumor effects: (1) Combined drugs target the inhibition sites of oncolytic viruses; (2) Genetic modification of oncolytic virus; (3) Select the appropriate strain. In addition, for tumor types with IFN deficiency, some specific defects in the IFN signaling cascade can be used as potential biomarkers, which may help identify such individual cancer patients and obtain personalized treatment (16).

Of course, there are still some problems in the study of the mechanism of action between oncolytic virus and IFNs. First, most studies are still in the preclinical stage, and their mechanisms and effects in the clinical setting are still unknown. Researchers should accelerate their studies to better benefit oncology patients. Second, due to the complexity of *in vivo* immunity, the relationship between the efficacy of viral tumor lysis and the IFNs gene has not been fully elucidated. Further studies in this area are expected in the future. In addition, many studies have used the expression level of IFNs as an indicator of antitumor effects, and the specific mechanism of its increased expression level and antitumor effects need to be further elucidated. Notably, there are few studies on type III IFN and oncolytic viruses, and it is hoped that future studies will fill this gap. Finally, the degree of research on various viruses varies greatly. Some viruses are able to activate IFN-induced antitumor immunity while avoiding immune clearance, such as PVSRIPO and HSV-1, etc. Such viruses may have more advantages in antitumor, and research on their oncolytic mechanism can be more widely carried out.

## Author contributions

QL and FT: writing, editing, and visualization. YW and XL: reviewing and editing. XK: conceptualization. JM: validation. LY: supervision. SC: funding acquisition. All authors contributed to the article and approved the submitted version.

## Funding

This study was supported by the Scientific research project of Tianjin Education Commission (2021KJ134), National Natural Science Foundation of China (81973728) and Science and Technology Program of Tianjin (21ZYJDJC00070).

## Acknowledgments

The figures were drawn by Figdraw (www.figdraw.com).

## Conflict of interest

The authors declare that the research was conducted in the absence of any commercial or financial relationships that could be construed as a potential conflict of interest.

## Publisher’s note

All claims expressed in this article are solely those of the authors and do not necessarily represent those of their affiliated organizations, or those of the publisher, the editors and the reviewers. Any product that may be evaluated in this article, or claim that may be made by its manufacturer, is not guaranteed or endorsed by the publisher.
